# Enhancement of ferromagnetism by oxygen isotope substitution in strontium ruthenate SrRuO_3_

**DOI:** 10.1038/srep35150

**Published:** 2016-10-14

**Authors:** Hirofumi Kawanaka, Yoshihiro Aiura, Takayuki Hasebe, Makoto Yokoyama, Takahiko Masui, Yoshikazu Nishihara, Takashi Yanagisawa

**Affiliations:** 1 Electronics and Photonics Research Institute, National Institute of Advanced Industrial Science and Technology (AIST), Central 2, 1-1-1 Umezono, Tsukuba, Ibaraki 305-8568, Japan; 2 Faculty of Science, Ibaraki University, 2-1-1 Bunkyo, Mito 310-8512, Japan; 3 Department of Physics, Kindai University, 3-4-1 Kowakae, Higashiosaka, Osaka 577-8502, Japan

## Abstract

The oxygen isotope effect of the ferromagnetic transition in itinerant ferromagnet strontium ruthenate SrRuO_3_ with a Curie temperature *T*_c_ of 160 K is studied. We observed for the first time a shift of ∆*T*_c_ ~ 1 K by oxygen isotope substitution of ^16^O → ^18^O in SrRuO_3_ by precise measurements of DC and AC magnetizations. The results surprisingly lead to the noteworthy inverse isotope effect with negative coefficient α = −∂ ln*T*_c_/∂ ln*M*. The Raman spectra indicate that the main vibration frequency of ^16^O at 363 cm^−1^ shifts to 341 cm^−1^ following oxygen isotope substitution ^18^O. This shift is remarkably consistent with the Debye frequency being proportional to ∝ 1√*M* where *M* is the mass of an oxygen atom. The positive isotope shift of ∆*T*_c_ can be understood by taking account of the electron-phonon interaction.

Strongly correlated electron systems (SCES) exhibit many interesting quantum phenomena such as unconventional superconductivity and metal–insulator transitions. SCES include high-temperature superconductors^1^,^2^,^3^,^4^, heavy fermions^5^,^6^,^7^,^8^, and organic conductors^9^. Electron–phonon interactions are important in metals and SCES. An unconventional isotope effect has been reported for high-temperature cuprate superconductors^10^,^11^. Electron–phonon interactions are ubiquitous in materials so it is important to investigate the role of electron–phonon coupling in SCES.

The isotope effect of the ferromagnetic transition in La_1−*x*_Ca_*x*_MnO_3_ has been investigated^12^,^13^. A large oxygen isotope effect was observed for La_1−*x*_Ca_*x*_MnO_3_ upon ^18^O substitution with the largest Curie Temperature (*T*_c_) shift of *T*_c_(^16^O) = 222.7 K to *T*_c_(^18^O) = 202.0 K observed when *x* = 0.20. This shift may be caused by strong electron–lattice coupling and has some relation with large magnetoresistance^14^,^15^. It has been suggested that the ferromagnetic transition of La_1−*x*_Ca_*x*_MnO_3_ is caused by the double-exchange interaction^16^,^17^,^18^ and its strong electron–lattice interaction originating from the Jahn–Teller effect^19^.

The isotope effect in the ferromagnetic insulating state of Pr_1−*x*_Ca_*x*_MnO_3_, which is also a material that exhibits colossal magnetoresistance, has been investigated^20^. The *T*_c_ of Pr_1−*x*_Ca_*x*_MnO_3_ was lowered upon isotope substitution ^16^O → ^18^O; for example, *T*_c_(^16^O) = 112 K shifted to *T*_c_(^18^O) = 106 K when *x* = 0.2. It is expected that this isotope effect arises from strong electron–phonon coupling^21^,^22^. The effect of isotope on *T*_c_ has also been examined for the ferromagnetic superconductor RuSr_2_GdCu_2_O_8_^23^. A small decrease of *T*_c_ of ~0.35 K was obtained through the isotope effect, which also influenced superconducting transition temperature. As for strontium ruthenates, an anomalous isotope effect was observed for the superconducting transition temperature of the spin-triplet superconductor Sr_2_RuO_4_^24^. Raman spectra of SrRuO_3_ films showed anomalous temperature dependence near the ferromagnetic transition temperature^25^. This indicates that the electron–phonon interaction plays a role in the ferromagnetism of SrRuO_3_. There has been an attempt to measure the isotope effect in the weak itinerant ferromagnet ZrZn_2_^26^. However, the isotope effect of *T*_c_ was not determined because the shift of *T*_c_ was small and there was uncertainty arising from different impurity levels. To date, a distinct isotope effect of itinerant ferromagnets has not been observed.

In this article, we report the isotope effect of *T*_c_ of the itinerant ferromagnet SrRuO_3_, which is a metal^27^,^28^ and with *T*_c_ of ~160 K^29^,^30^. We observe for the first time that the ferromagnetic transition temperature of SrRuO_3_ is increased about 1 K upon ^18^O isotope substitution. This positive isotope effect needs a new analogy to explain the ferromagnetic interaction in itinerant electron systems. A softening of the oxygen vibration modes is induced by isotope substitution (^16^O → ^18^O), which is clearly indicated by Raman spectroscopy. The Raman spectra also confirm that almost all the oxygen atoms (more than 80%) are substituted.

The increase of atomic mass caused by isotope substitution leads to a decrease of the Debye frequency *ω*_D_. In fact, the Raman spectra of SrRuO_3_ before and after oxygen isotope substitution clearly indicate that the main vibration frequency of ^16^O at 372 cm^*−*1^ is lowered to 351 cm^*−*1^ for ^18^O. This shift is consistent with *ω*_D_ being proportional to 1*/*√*M*, where *M* is the mass of an oxygen atom. Thus, our experiments confirm that the isotope shift of *T*_c_ is induced by the decrease of the frequency of the oxygen vibration mode. Our results reveal a small increase of *T*_c_ on ^18^O substitution, which is in contrast to the typical oxygen isotope effects of materials showing colossal magnetoresistance. The oxygen isotope effect in SrRuO_3_ arises from the electron–phonon interaction described by the electron–phonon coupled field theory^31^ because there is no lattice distortion such as the Jahn–Teller effect. We present a theory to account for the isotope effect with the positive shift of *T*_c_ in itinerant ferromagnets by considering the spin-fluctuation theory for ferromagnets^32^. The decrease of *ω*_D_ increases the relative strength of the Coulomb interaction *U*. This results in a positive isotope shift of *T*_c_ in accordance with our experimental results.

## Experiment

**Figure 1:**
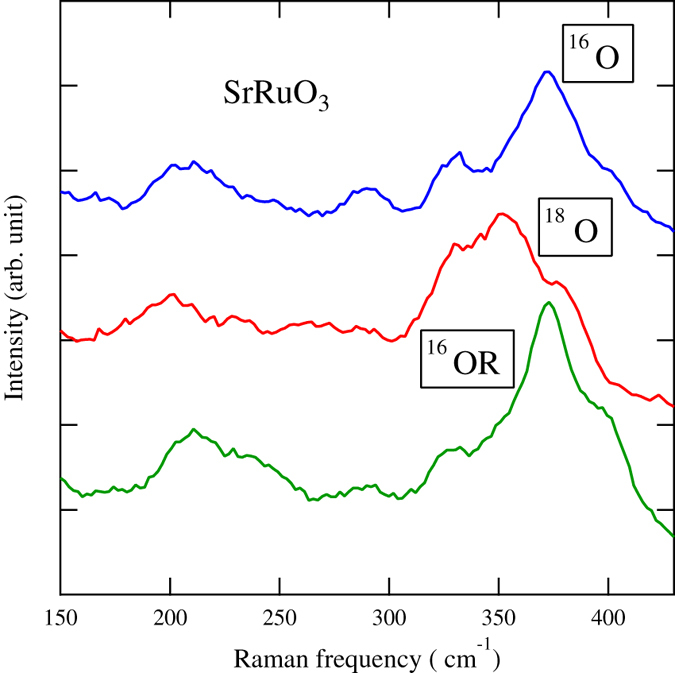
Intensity of Raman scattering as a function of frequency for SrRuO_3_ samples with different oxygen isotopes at room temperature.

The matrix of SrRuO_3_ was synthesized by the conventional solid-state reaction method from a stoichiometric mixture of SrCO_3_ and RuO_2_. The starting materials were calcined at 800 °C for 24 h and then sintered at 1000 °C for 48 h in air, resulting in a dense pellet. Oxygen isotope substitution was performed by annealing. To ensure oxygen isotope substitution, samples were first annealed under the following conditions. Samples were places in two independent quartz tubes in a furnace and exposed to ^16^O_2_ or ^18^O_2_ gas at atmospheric pressure. Samples in both ^16^O_2_ and ^18^O_2_ gas were annealed at 1000 °C for 91 h, cooled in the furnace to room temperature and then annealed again at 1000 °C for 90 h. Raman spectroscopy of the samples confirmed that >80% of the oxygen atoms were substituted (Fig. 1).

DC and AC magnetization measurements were conducted to determine *T*_c_. DC magnetization measurements were carried out by a superconducting quantum interference device magnetometer (Quantum Design Inc., MPMS2) up to 5 Tesla at temperatures *T* from 5 to 300 K. AC magnetic susceptibility was measured using a Hartshorn bridge circuit. The AC magnetic field produced by the primary coil was 1 Oe and the frequency of the AC signal was 180 Hz.

The oxygen phonon modes are observed at room temperature. A softening of the oxygen vibration modes was confirmed by Raman spectroscopy. We resubstituted an ^18^O-substituted sample with ^16^O to produce a sample indicated as ^16^OR. The Raman spectrum of ^16^OR is also shown in Fig. 1.

**Figure 2:**
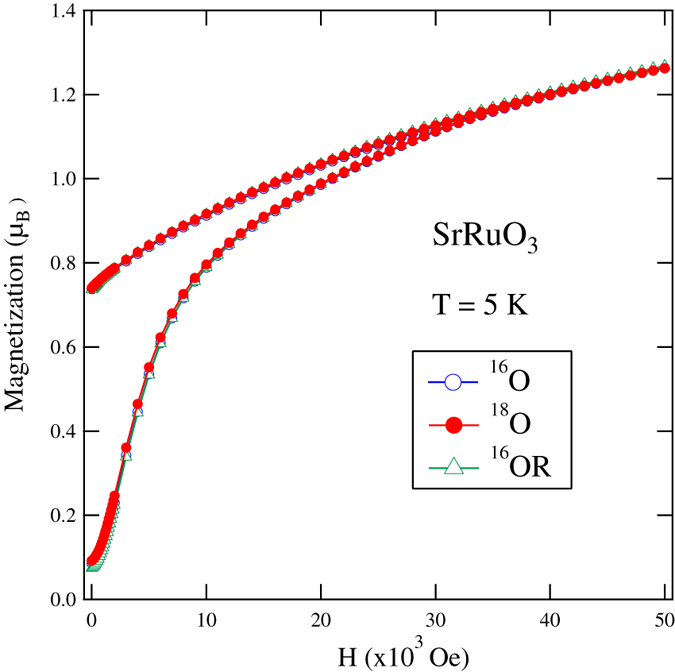
Magnetization as a function of magnetic field for SrRuO_3_ samples with different oxygen isotopes at temperature *T* = 5 K.

The magnetic field dependence of magnetization of SrRuO_3_ samples substituted with oxygen ^16^O, ^18^O, and ^16^OR at *T* = 5 K are presented in Fig. 2. These three magnetization curves show almost the same magnetization process.

**Figure 3:**
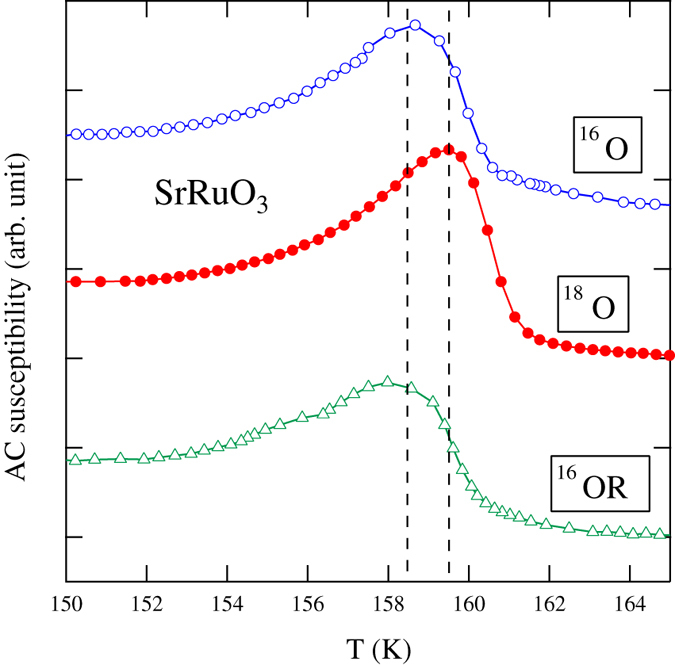
AC magnetic susceptibility as a function of temperature *T* for SrRuO_3_ samples with different oxygen isotopes.

**Figure 4:**
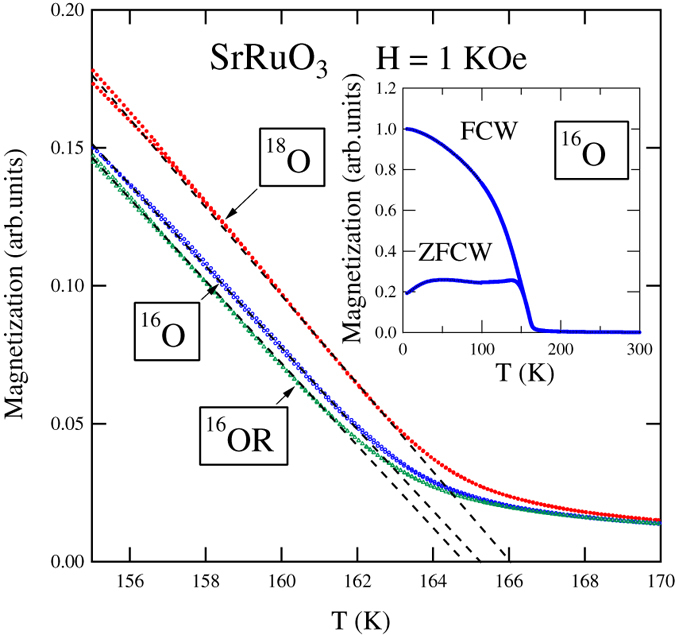
Magnetization as a function of temperature *T* for the original SrRuO_3_ sample with ^16^O under an applied magnetic field *H* = 1 kOe. Inset is the temperature dependence of magnetization of SrRuO_3_ following oxygen isotope substitution with ^16^O at *H* = 1 kOe.

The temperature dependences of AC magnetic susceptibility of SrRuO_3_ samples substituted with ^16^O, ^18^O and ^16^OR are illustrated in Fig. 3. A shift of peak position caused by ^18^O substitution is clearly observed. The shift shows that ∆*T*_c_ is ~1 K. Therefore, the ferromagnetic transition temperature of SrRuO_3_ was increased by about 1 K by ^18^O substitution. The temperature dependence of magnetization at 1 kOe for SrRuO_3_ following oxygen isotope substitution is depicted in Fig. 4. The ferromagnetic transition temperature of SrRuO_3_ is increased by ^18^O substitution. Again, the shift indicates that ∆*T*_c_ is ~1 K. This agrees with the shift estimated from the AC magnetic susceptibility measurements.

## Disscussion

**Figure 5:**
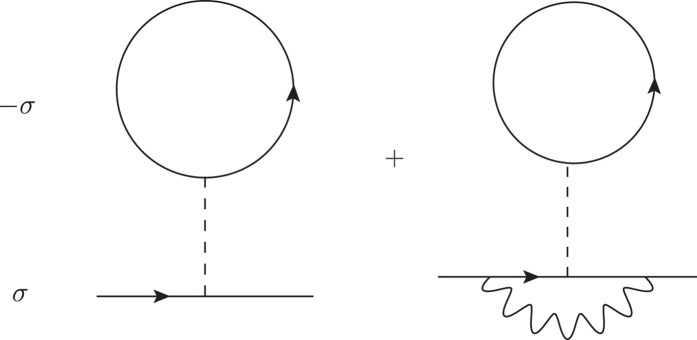
Lowest-order electron self-energy corrections. The second term arises from the electron–phonon vertex correction. The dashed line indicates the Coulomb interaction and the wavy line shows the phonon propagator.

**Figure 6:**
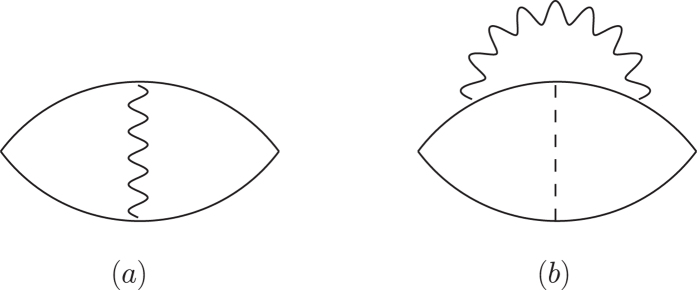
Contributions to the susceptibility *χ*^+*−*^ (**a**) without and (**b**) with the electron–phonon vertex correction.

Let us examine the isotope effect theoretically. An important point is that the decrease of *ω*_D_ increases the relative strength of the Coulomb interaction *U*. This leads to the positive shift of *T*c in accordance with experiments. This is stated theoretically as follows. In the study of magnetism, the Hubbard model is of fundamental importance^32^,^33^,^34^,^35^,^36^,^37^. The isotope effect in an itinerant ferromagnet was investigated before using the RPA theory^38^,^39^. We consider a model with Hubbard on-site Coulomb repulsion and electron–phonon interaction. *T*_c_ is determined through solution of the gap equation. The Hamiltonian is given by


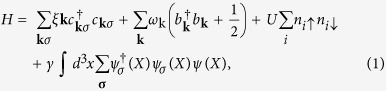


where *c*_**k***σ*_ and *c*^*†*^_**k***σ*_ are Fourier transforms of the annihilation and creation operators *c*_*iσ*_ and *c*^*†*^_*iσ*_ at site *i*, respectively. Here, *n*_*iσ*_ = *c*^*†*^_*iσ*_*c*_*iσ*_ is the number operator, and *ξ*_**k**_ = *ε*_**k**_ *− μ* is the dispersion relation measured from the chemical potential *μ*. The electron field *ψ*_*σ*_ and the phonon field *ϕ* are defined, respectively, as follows:


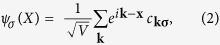



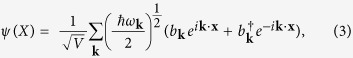


where *V* is the volume of the system. We denote the number of electrons with spin *σ* as *n*_*σ*_ = (1*/*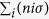, where *N* is the number of sites. In the mean-field theory, the magnetization is determined by 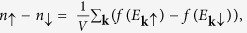
where *E*_**k***σ*_ is the electron dispersion relation including the corrections in Fig. 5, which are given as


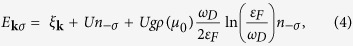


where *g* = *−γ*^2^, and *ρ*(*μ*_0_) is the density of states at the Fermi level. The vertex correction is of the order of *ω*_*D*_*/E*_*F*_ in accordance with the Migdal theorem^40^,^41^,^42^. For the magnetization ∆ ≡ *n*↑ *−* *n*↓, we obtain the equation up to the order of ∆ of, 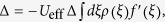 where *U*_eff_ = *U* + *Ugρ*(*μ*_0_)(*ω*_*D*_*/*2*ε*_*F*_)ln(*ε*_*F*_*/ω*_*D*_). With the help of the Sommerfeld expansion, *T*_c_ in the mean-field approximation is given by


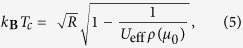


where *R* is a constant. It has been suggested that the vertex correction like that shown in Fig. 6(b) is important in evaluating *T*_c_ 39. We examined this by calculating the diagrams in Fig. 6(a,b) numerically, and found that the contribution in Fig. 6(b) is smaller than that in Fig. 6(a) by more than one order of magnitude. The sum of all particle–hole ladder diagrams will increase the term in Fig. 6(b), but this is restricted to a very small region just near the critical value of *U*. Thus, from the formula of *T*_c_ in Eq. (5) we obtain the positive isotope shift of *T*_c_: ∂*T*_c_*/*∂*M*** > **0. The positive isotope shift of *T*_c_ is also obtained by taking the spin fluctuation effect into account^32^,^43^,^44^. The isotope coefficient *α* = *−*∂ ln *T*_c_*/*∂ ln *M* is given as


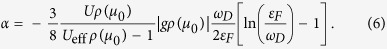


This indicates that α is negative and *T*c increases with *M*.

## Conclusion

We examined the isotope effect of *T*_c_ in itinerant ferromagnet SrRuO_3_. First, *T*_c_ was estimated from DC and AC magnetization measurements. We found that *T*_c_ increases upon oxygen isotope substitution of ^16^O for ^18^O. This results in an inverse isotope effect of ∂ ln *T*_c_ */*∂ ln *M* < 0. This finding is in contrast to the results obtained for Mn oxides that show colossal magnetoresistance. The isotope effect of *T*_c_ occurs through the electron–phonon interactions, especially the electron–phonon vertex correction.

We summarize the results and significance of our work as follows. (1) The inverse isotope effect of the Curie temperature was observed clearly in an itinerant ferromagnetic material for the first time. (2) Our result shows that the electron-phonon interaction is ubiquitous in the world, even in ferromagnetic materials. (3) The positive shift of the Curie temperature can be understood based on a theoretical model of the Coulomb interaction and the electron-phonon interaction. (4) We have established the experimental procedure to substitute isotope oxygen ^18^O for ^16^O. This technique would be helpful in searching new functional materials.

## Additional Information

**How to cite this article**: Kawanaka, H. *et al*. Enhancement of ferromagnetism by oxygen isotope substitution in strontium ruthenate SrRuO_3_. *Sci. Rep*. **6**, 35150; doi: 10.1038/srep35150 (2016).
